# Heatstroke Awareness and Preventive Behaviors Among Automotive Maintenance Workers in Outdoor Environments: A Cross-Sectional Study in Japan

**DOI:** 10.3390/healthcare14101293

**Published:** 2026-05-10

**Authors:** Chieko Yodawara, Yoko Iio, Harumi Ejiri, Saimi Yamamoto, Hana Kozai, Mamoru Tanaka, Manato Seguchi, Morihiro Ito

**Affiliations:** 1 Graduate School of Life and Health Sciences, Chubu University, 1200 Matsumoto-cho, Kasugai-shi 487-8501, Japan; 2 Department of Nursing, Nagoya University of Arts and Sciences, 4-1-1 Sannomaru, Naka-ku, Nagoya-shi 460-0001, Japan; 3 Department of Lifelong Sports and Health Sciences, College of Life and Health Sciences, Chubu University, 1200 Matsumoto-cho, Kasugai-shi 487-8501, Japan; 4 Department of Nursing, College of Life and Health Sciences, Chubu University, 1200 Matsumoto-cho, Kasugai-shi 487-8501, Japan; 5 Department of Early Childhood Education, College of Contemporary Education, Chubu University, 1200 Matsumoto-cho, Kasugai-shi 487-8501, Japan; 6 Department of Food and Nutritional Sciences, College of Bioscience and Biotechnology, Chubu University, 1200 Matsumoto-cho, Kasugai-shi 487-8501, Japan; 7 Support for Pioneering Research Initiated by the Next Generation (SPRING), Chubu University, 1200 Matsumoto-cho, Kasugai-shi 487-8501, Japan; 8 Department of Biomedical Sciences, College of Life and Health Sciences, Chubu University, 1200 Matsumoto-cho, Kasugai-shi 487-8501, Japan

**Keywords:** heatstroke, occupational heat stress, automotive maintenance workers, preventive behavior, risk perception, Japan

## Abstract

**Background/Objectives:** Global climate change has increased occupational heat exposure, posing significant risks to outdoor workers. Automotive maintenance workers face high temperatures, radiant heat from machinery, and physically demanding tasks; however, their awareness and preventive behaviors regarding heat-related illness remain insufficiently understood. This study examined heatstroke awareness and preventive behaviors among automotive maintenance workers in Japan. **Methods:** A cross-sectional web-based survey was conducted among 371 automotive maintenance workers. Self-reported heat-related illness experience was assessed based on subjective judgment without formal medical diagnosis. Associations between heat-related illness experience and behavioral, physical, and health-related factors were analyzed using chi-square tests with Bonferroni correction and multivariable logistic regression. **Results:** Approximately 39.6% of participants reported experiencing heat-related illness during summer work. In multivariable analysis, headache (OR: 2.66, 95% CI: 1.25–5.64), dizziness (OR: 2.06, 95% CI: 1.12–3.80), obesity (OR: 1.86, 95% CI: 1.06–3.27), and lower self-perceived health (OR: 2.19, 95% CI: 1.36–3.55) were independently associated with heat-related illness experience. Some preventive behaviors, including wearing cooling garments and frequent hydration, showed associations in the multivariable analysis; however, these findings should be interpreted with caution due to possible reverse causation, small cell sizes, and residual confounding. **Conclusions:** Behavioral and individual health-related factors, particularly symptom recognition and self-perceived health, are associated with heat-related illness experience among automotive maintenance workers. Interventions focusing on early symptom awareness, risk perception, and self-monitoring may be important components of workplace-based heat illness prevention. Future studies incorporating objective environmental and physiological measurements are needed to clarify causal relationships.

## 1. Introduction

Global climate change has led to a steady increase in ambient temperatures, intensifying occupational heat exposure worldwide. Outdoor workers are particularly vulnerable to heat-related illnesses, including heatstroke, due to prolonged exposure to high temperatures and physically demanding tasks. Heatstroke is a serious condition characterized by elevated core body temperature and central nervous system dysfunction, potentially leading to severe outcomes or death if not promptly managed. In recent years, increasing attention has been paid to the impact of heat stress on occupational safety, health, and productivity [1,2,3,4].

Among outdoor workers, automotive maintenance workers represent a unique and understudied population. In addition to environmental heat exposure, these workers are exposed to radiant heat from engines and machinery, as well as confined workspaces that can trap heat and humidity. Their work often involves physically demanding tasks, which further increase internal heat production. Despite these risk factors, little is known about how automotive maintenance workers perceive heat-related risks or how they engage in preventive behaviors.

Occupational heat stress has been extensively studied in several high-risk industries. Construction workers are among the most affected groups, second only to agricultural workers, with heat-related illness symptoms reported in approximately 36% of workers across multiple geographic settings [5]. Agricultural workers face similarly elevated risks due to prolonged outdoor exposure and high physical workload [6]. Other sectors, including mining, logistics, manufacturing, and military settings, have also been identified as occupationally vulnerable to heat-related illness [7]. Despite this growing body of evidence across sectors, research on automotive maintenance workers—who face a unique combination of radiant heat from machinery, confined workspaces, and physically demanding tasks—remains scarce.

Previous studies have identified various physiological and environmental risk factors for heatstroke, including high ambient temperature, excessive physical exertion, obesity, inadequate hydration, and insufficient heat acclimatization [8,9,10,11,12,13,14]. However, these studies have primarily focused on environmental or physiological determinants, with limited attention given to behavioral factors. From a behavioral science perspective, individual awareness, risk perception, and self-regulation behaviors play a critical role in preventing heat-related illnesses. The ability to recognize early symptoms, assess personal risk, and take appropriate preventive actions may significantly influence outcomes in high-risk environments [12,13].

Behavioral factors are particularly important in the context of heatstroke, as early symptoms such as headache and dizziness often precede severe conditions. Timely recognition of these symptoms and appropriate behavioral responses, such as resting, cooling, or hydration, are essential to prevent progression to severe heat illness [15]. However, the relationship between workers’ awareness, preventive behaviors, and actual heatstroke experience remains insufficiently understood, especially in specific occupational settings.

Furthermore, preventive measures implemented by workers may not always lead to the intended outcomes. In some cases, individuals who have previously experienced heatstroke may adopt more preventive behaviors, which can complicate the interpretation of associations between behavior and health outcomes [16]. Understanding these behavioral patterns requires careful consideration of both risk perception and prior experience. This study addresses an important gap in the literature by focusing on a high-risk yet understudied occupational group.

Given these gaps, this study aimed to examine heatstroke awareness and preventive behaviors among automotive maintenance workers engaged in outdoor work in Japan, from a behavioral science perspective. Specifically, this study investigated the associations between self-reported heatstroke experience and behavioral, physical, and health-related factors. By focusing on behavioral determinants such as awareness, symptom recognition, and preventive actions, this study seeks to provide insights that can inform the development of effective educational and behavioral interventions for heatstroke prevention in occupational settings.

## 2. Materials and Methods

### 2.1. Study Design and Data Collection

A cross-sectional study was conducted in Japan from late March to early April 2025 using an anonymous, self-administered questionnaire survey targeting automotive maintenance workers engaged in outdoor work. The study aimed to assess their awareness of heatstroke and their adaptive behaviors during summer work.

Data collection used a web-based questionnaire administered through Google Forms (anonymous survey), which included 31 items. Workers who consented accessed the survey by scanning the Google Form URL QR code with their mobile phones. By the end of April 2025, researchers collected 494 responses. Of these, 371 valid responses (75.1% response rate) were analyzed. Responses were excluded if participants did not consent to participation, submitted incomplete questionnaires, or did not meet the eligibility criteria of being engaged in outdoor automotive maintenance work. Participants were recruited through their workplaces via QR codes distributed by the research team. The survey targeted workers across multiple regions of Japan, and no financial incentives were provided for participation.

To determine the required sample size for this study, statistical power analysis was conducted using Statistical Package for Social Sciences, version 29, (SPSS; IBM Corp., Armonk, NY, USA). A sample size of 202 was needed to achieve a power of 0.8 and a significance level of 0.05, based on the number of explanatory variables included in the regression model. The sample size used in this study met these criteria.

This study followed the principles of the Declaration of Helsinki and received approval from the Chubu University Ethics Review Board (Approval No. 20240091). Participants received a written explanation outlining the study’s purpose, the voluntary nature of participation, assurance that non-participation would not affect their work, confirmation that results would be used only for the stated purpose, and that the study was anonymous with no identifiable individuals. The explanation also addressed compliance with personal information protection, including data management. Consent was considered obtained when participants completed the questionnaire.

### 2.2. Survey Items

The survey collected data on basic attributes (sex, age group, and region of workplace), past experiences of heatstroke during work activities, physical symptoms of heatstroke during work and preventive measures, experiences of receiving heatstroke education, lifestyle conditions such as diet and sleep, and health status. For questions about physical symptoms, 13 symptoms were defined according to the Japanese Association for Acute Medicine’s classification of heatstroke and previous research [17]. Table S1 presents a comprehensive overview of all variables used in the analysis, including the corresponding survey items, response options, and coding procedures. 

### 2.3. Statistical Analysis

The collected data were organized using simple tabulation. We hypothesized that self-reported heatstroke experience during work is associated with various living environments and health conditions. To test this, we analyzed the relationship between participants’ heatstroke experience and preventive measures, lifestyle habits, dietary intake, and health status using Pearson’s chi-square test. Variables that reached statistical significance in the chi-square tests were entered into a binary logistic regression analysis to identify factors associated with heatstroke experience. Variable selection was based on both statistical significance and theoretical relevance informed by prior literature on occupational heat stress. Key variables such as headache, dizziness, BMI, and perceived health were included a priori based on prior literature, regardless of their statistical significance in bivariate analysis. Multicollinearity among explanatory variables was assessed using the variance inflation factor (VIF), and no variables exceeded the threshold of VIF > 10. Model fit was evaluated using the Hosmer–Lemeshow test and Nagelkerke R^2^. Age and sex were included as potential confounders in the model.

The dependent variable was the presence or absence of heatstroke experience. Explanatory variables included physical symptoms during summer work activities, heatstroke prevention measures, living conditions, and health status. Table 1 shows basic attributes. In Table 2, dummy variables were coded as “None” = 0 and “Present” = 1. In Table 3, dummy variables were coded as “Present” = 0 and “None” = 1. For Table 4-A, explanatory variables were coded as dummy variables: “Normal (body mass index [BMI] between 18 and <25)” = 0, “Underweight (<18.5)” = 1, and “Obese (≥25)” = 2. In Table 4-B, explanatory variables were coded as “Normal, Underweight (<18.5)” = 0 and “Obese (≥25)” = 1. Explanatory variables in Table 4-K and L were coded as “Yes” = 0 and “No/Neither” = 1. Odds ratios (OR) and 95% confidence intervals (CI) were calculated for each explanatory variable. The significance level was set at *p* < 0.05 for all items. To control for the inflated risk of Type I error due to multiple comparisons, the Bonferroni correction was applied to the chi-square tests in Table 2, Table 3 and Table 4. The corrected significance thresholds were set at *p* < 0.0038 (Table 2, 13 comparisons), *p* < 0.0020 (Table 3, 25 comparisons), and *p* < 0.0026 (Table 4, 19 comparisons). The logistic regression analysis was not subject to this correction, as the simultaneous inclusion of variables in the model inherently adjusts for their intercorrelations. SPSS statistical analysis software was used for the analysis.

## 3. Results

### 3.1. Participant Attributes

Table 1 presents the relationship between heatstroke experience and participant attributes. Of the 371 workers, 147 (39.6%) reported experiencing heatstroke during summer work, while 224 (60.4%) did not. There were 302 men (81.4%) and 69 women (18.6%). Among these, 112 men (37.1%) and 35 women (50.7%) reported heatstroke experience, indicating that a higher proportion of women reported experiencing heatstroke compared to men.

The largest age group was 20–29 years, with 243 respondents (65.5%). The next largest groups were 30–39 years (77 respondents, 20.8%), 40–49 years (30 respondents, 8.1%), 50–59 years (11 respondents, 2.9%), and 10–19 years (10 respondents, 2.7%). Among respondents who reported experiencing heatstroke, the largest group was 30–39 years (38 respondents, 49.4%). Age was not significantly associated with self-reported heatstroke experience.

Most respondents worked in the “Kanto region” (eastern Japan), with 142 respondents (38.3%), and the “Chubu region” (central Japan), with 138 respondents (37.2%). The “Kinki region” (western Japan) had 45 respondents (12.1%), the Chugoku/Shikoku region had 13 respondents (3.5%), the “Kyushu region” had 12 respondents (3.2%), the “Hokkaido region” in northern Japan had 11 respondents (3.0%), and the “Tohoku region” had 10 respondents (2.7%). There was no correlation between region of employment and self-reported experience of heatstroke.

### 3.2. Physical Symptoms

Table 2 presents the relationship between heatstroke experience during summer work and physical symptoms. Workers who reported experiencing heatstroke during summer work more frequently reported symptoms such as “headache,” “dry mouth,” “dizziness,” “fatigue/weakness,” “difficulty concentrating/inability to organize thoughts,” “nausea,” “muscle cramps,” “abnormally rapid breathing,” “rapid and weak pulse,” “numbness in the lips,” and “abnormal speech or behavior” compared to those who did not experience heatstroke.

### 3.3. Preventive Measures

Table 3 presents the relationship between heatstroke experience and preventive measures. Although a higher proportion of workers who reported wearing cooling fabric underwear indicated having experienced heatstroke in the univariate analysis (*p* = 0.024), this association did not survive Bonferroni correction (threshold *p* < 0.002) and should be interpreted with caution. This finding may reflect reverse causation, whereby workers who had previously experienced heatstroke were more likely to adopt preventive behaviors thereafter.

Regarding frequent hydration, 352 respondents (94.9%) reported practicing it. The cell size for non-hydrators who reported heatstroke experience was extremely small (n = 2), and this result should therefore be interpreted with extreme caution.

There was no correlation between self-reported heatstroke experience and responses about “use of heat index (WBGT),” “heat acclimatization,” “experience viewing the Ministry of Health, Labour and Welfare’s Heatstroke Prevention Manual,” or “experience receiving education or guidance on heatstroke countermeasures.”

### 3.4. Living Conditions and Health Status

Table 4 presents the relationship between heatstroke experience and living conditions or health status. For BMI, workers classified as obese (≥25) reported a higher rate of heatstroke experience compared to those classified as normal or underweight (<18.5).

Workers who reported “yes” for “satisfaction with sleep” and “perceived health” had a lower proportion of “experienced heatstroke” compared to those who answered “no” or “neither yes nor no.”

There was no relationship between self-reported heatstroke experience and factors such as food intake, sleep duration, alcohol consumption, smoking status, hypertension, dyslipidemia, illness or treatment, or medication use.

### 3.5. Factors Contributing to Heatstroke Experience

Table 5 presents the results of the binary logistic regression analysis of factors associated with heatstroke experience. Experiencing headaches during summer work (OR: 2.657, 95% CI: 1.253–5.636, *p* = 0.011) and experiencing dizziness during summer work (OR: 2.061, CI: 1.118–3.800, *p* = 0.021) were symptoms linked to self-reported heatstroke. Higher BMI (OR: 1.857, CI: 1.056–3.267, *p* = 0.032) and self-perceived health status (OR: 2.194, CI: 1.355–3.551, *p* = 0.001) were significantly associated with heatstroke experience. In contrast, wearing cooling fabric underwear (OR: 0.590, CI: 0.370–0.939, *p* = 0.026) and frequent hydration (OR: 0.187, CI: 0.041–0.863, *p* = 0.032) showed associations in the multivariable analysis; however, these findings should be interpreted with caution due to possible reverse causation, small cell sizes, and residual confounding.

## 4. Discussion

This study examined heatstroke awareness and preventive behaviors among automotive maintenance workers engaged in outdoor work in Japan from a behavioral science perspective. The findings provide novel insights into how behavioral, physical, and health-related factors are associated with self-reported heatstroke experience in a high-risk occupational setting.

Approximately 40% of workers reported experiencing heatstroke during summer work, indicating a substantial burden of heat-related illness in this population. This prevalence appears higher than that reported in some previous occupational studies, suggesting that automotive maintenance workers may be exposed to particularly hazardous thermal environments. In addition to ambient heat, exposure to radiant heat from engines and confined workspaces likely contributes to cumulative heat stress, emphasizing the need for targeted preventive strategies [2,3,4,18].

From a behavioral science perspective, one of the most important findings of this study is the significant association between early symptoms—such as headache and dizziness—and heatstroke experience. These symptoms are generally considered early warning signs of heat-related illness, and their recognition plays a critical role in preventing progression to severe heatstroke. The results suggest that workers who experience these symptoms may either be at higher risk or may become more aware of heat-related physiological changes. Therefore, enhancing symptom awareness and promoting early behavioral responses, such as rest and cooling, are likely to be key components of effective heatstroke prevention strategies [15,17].

In the logistic regression analysis, certain preventive behaviors, including wearing cooling fabric underwear and frequent hydration, showed inverse associations with self-reported heatstroke experience after adjustment for confounders. This suggests that, once the influence of factors such as obesity and poor self-perceived health is statistically controlled, workers who engaged in these behaviors tended to report fewer heatstroke experiences—yet this pattern alone does not confirm a protective effect, given the cross-sectional design. However, neither association survived Bonferroni correction in the univariate chi-square analysis. The discrepancy between the univariate and multivariate findings is consistent with reverse causation—workers who had previously experienced heatstroke may have subsequently increased their use of preventive measures, inflating the apparent association in the univariate analysis. These findings should therefore be interpreted with caution, and longitudinal studies are needed to establish the causal direction of these relationships. In particular, the finding regarding frequent hydration should be considered exploratory only, given the extremely small cell size in the non-hydration group (n = 2 with heatstroke experience), the failure to survive Bonferroni correction, and the wide confidence interval observed in the logistic regression (OR: 0.187, 95% CI: 0.041–0.863). This result should not be interpreted as evidence of a protective effect of hydration.

In addition, preventive behaviors may not always be implemented appropriately or effectively. For example, wearing cooling garments under multiple layers of protective clothing may limit heat dissipation, and hydration practices may vary in timing, volume, and composition. These findings suggest that not only the presence of preventive behaviors, but also their quality and appropriateness, are critical. From a behavioral science standpoint, this underscores the importance of not only promoting preventive actions but also improving knowledge and decision-making related to those actions [12,13].

The present study also identified obesity (BMI ≥ 25) as a factor associated with heatstroke experience, which is consistent with previous studies indicating that higher body fat may impair heat dissipation and increase thermal strain [19,20,21]. In contrast, higher self-perceived health status was associated with a lower likelihood of heatstroke. This finding suggests that subjective health perception may reflect underlying physical condition, lifestyle habits, or self-regulation capacity. Individuals who perceive themselves as healthy may be more likely to engage in adaptive behaviors, monitor their condition, and respond appropriately to heat stress.

Importantly, this study highlights the role of behavioral factors such as risk perception, awareness, and self-monitoring in heatstroke prevention. Workers’ ability to recognize their own vulnerability, interpret physiological signals, and take timely action is central to preventing adverse outcomes. These findings support the need for behavioral interventions that enhance risk perception and promote adaptive decision-making in high-risk environments. Educational programs should focus not only on knowledge dissemination but also on improving practical skills, such as symptom recognition and appropriate response behaviors [12,13].

From an occupational health perspective, these findings have important implications for workplace interventions. For example, incorporating routine health checks, promoting open communication about physical symptoms, and implementing structured education programs may improve early detection and prevention of heatstroke. In addition, environmental and organizational measures—such as monitoring heat indices (e.g., WBGT), adjusting work–rest cycles, and optimizing clothing strategies—should be integrated with behavioral approaches to achieve comprehensive risk management [2,3,4,18].

This study has several limitations. First, the cross-sectional design precludes causal inference, and reverse causation cannot be ruled out. Second, heatstroke experience was self-reported and may be subject to recall bias or misclassification. Third, the sample was predominantly male (81.4%), which is consistent with the occupational distribution of automotive maintenance workers in Japan, where males represent the vast majority of the workforce. The number of female participants (n = 69) may therefore reflect an overrepresentation relative to the general population of this occupation. Accordingly, sex-specific findings should be interpreted in light of this occupational context, and future studies with larger female samples are warranted. Fourth, detailed information on hydration practices, clothing characteristics, and environmental heat exposure was not collected. Objective workplace measurements such as temperature, humidity, wet-bulb globe temperature (WBGT), and working hours were not obtained. Future studies should incorporate meteorological and physiological data to better characterize occupational heat exposure. Fifth, the questionnaire was developed by the research team based on prior literature and established clinical classifications, without formal psychometric validation or pilot testing. This may have affected the reliability and validity of individual items, and future studies should employ formally validated instruments. Finally, the use of a web-based survey may have introduced selection bias. Future studies should employ longitudinal designs, objective physiological measurements, and detailed behavioral assessments to better understand causal relationships and refine intervention strategies.

Despite these limitations, this study provides important contributions by focusing on a high-risk yet understudied occupational group and by examining heatstroke from a behavioral science perspective. The findings suggest that improving awareness, risk perception, and self-regulation behaviors may play a crucial role in preventing heat-related illness. Integrating behavioral interventions with occupational health management strategies may enhance the effectiveness of heatstroke prevention in real-world work environments.

## 5. Conclusions

This study demonstrated that behavioral factors, including symptom recognition, risk perception, and self-monitoring, are significantly associated with heatstroke experience among automotive maintenance workers. Preventive behaviors such as frequent hydration and the use of cooling garments showed associations with self-reported heatstroke experience; however, these findings should be interpreted cautiously due to potential reverse causation and study design limitations. These findings highlight the importance of behavior-based strategies in occupational heatstroke prevention. Future interventions should focus on improving awareness, decision-making, and appropriate behavioral responses in high-risk work environments. These findings have important implications for occupational health and workplace safety management.

## Figures and Tables

**Table 1:** Relationship between heatstroke experience and participant attributes.

		Heatstroke Experience						*p*-Value	
		No		Yes					
		n	(%)	n	(%)	n	(%)		
Question		224	(60.4)	147	(39.6)	371	(100.0)		
Sex	Male	190	(62.9)	112	(37.1)	302	(81.4)	0.037	*
	Female	34	(49.3)	35	(50.7)	69	(18.6)		
Age	10–19	9	(90.0)	1	(10.0)	10	(2.7)	0.095	
	20–29	151	(62.1)	92	(37.9)	243	(65.5)		
	30–39	39	(50.6)	38	(49.4)	77	(20.8)		
	40–49	17	(56.7)	13	(43.3)	30	(8.1)		
	50–59	8	(72.7)	3	(27.3)	11	(2.9)		
Region	Hokkaido Region	7	(63.6)	4	(36.4)	11	(3.0)	0.91	
	Tohoku Region	5	(50.0)	5	(50.0)	10	(2.7)		
	Kanto Region	86	(60.6)	56	(39.4)	142	(38.3)		
	Chubu Region	86	(62.3)	52	(37.7)	138	(37.2)		
	Kinki Region	24	(53.3)	21	(46.7)	45	(12.1)		
	Chugoku and Shikoku Region	9	(69.2)	4	(30.8)	13	(3.5)		
	Kyushu Region	7	(58.3)	5	(41.7)	12	(3.2)		

* *p* < 0.05.

**Table 2:** Relationship between heatstroke experience and physical symptoms during work activities.

		Heatstroke Experience						*p*-Value	
		No		Yes					
		n	(%)	n	(%)	n	(%)		
Question		224	(60.4)	147	(39.6)	371	(100.0)		
Headache	No (not at all)	71	(84.5)	13	(15.5)	84	(22.6)	<0.001	**
	Yes	153	(53.3)	134	(46.7)	287	(77.4)		
Dry mouth	No	40	(76.9)	12	(23.1)	52	(14.0)	0.009	**
	Yes	184	(57.7)	135	(42.3)	319	(86.0)		
Dizziness	No	99	(79.2)	26	(20.8)	125	(33.7)	<0.001	**
	Yes	125	(50.8)	121	(49.2)	246	(66.3)		
Fatigue/Weakness	No	64	(86.5)	10	(13.5)	74	(19.9)	<0.001	**
	Yes	160	(53.9)	137	(46.1)	297	(80.1)		
Difficulty concentrating/ Inability to organize thoughts	No	69	(81.2)	16	(18.8)	85	(22.9)	<0.001	**
	Yes	155	(54.2)	131	(45.8)	286	(77.1)		
Nausea	No	138	(72.3)	53	(27.7)	191	(51.5)	<0.001	**
	Yes	86	(47.8)	94	(52.2)	180	(48.5)		
Muscle cramps	No	138	(70.4)	58	(29.6)	196	(52.8)	<0.001	**
	Yes	86	(49.1)	89	(50.9)	175	(47.2)		
Abnormally rapid breathing	No	148	(69.8)	64	(30.2)	212	(57.1)	<0.001	**
	Yes	76	(47.8)	83	(52.2)	159	(42.9)		
Rapid and weak pulse	No	152	(69.4)	67	(30.6)	219	(59.0)	<0.001	**
	Yes	72	(47.4)	80	(52.6)	152	(41.0)		
Numbness in the lips	No	177	(65.1)	95	(34.9)	272	(73.3)	0.002	**
	Yes	47	(47.5)	52	(52.5)	99	(26.7)		
Abnormal speech and behavior	No	164	(64.3)	91	(35.7)	255	(68.7)	0.022	*
	Yes	60	(51.7)	56	(48.3)	116	(31.3)		
Fainting	No	193	(60.5)	126	(39.5)	319	(86.0)	0.904	
	Yes	31	(59.6)	21	(40.4)	52	(14.0)		
Hallucinations	No	188	(60.5)	123	(39.5)	311	(83.8)	0.948	
	Yes	36	(60.0)	24	(40.0)	60	(16.2)		

** *p* < 0.01; * *p* < 0.05.

**Table 3:** Relationship between heatstroke experience and preventive measures.

		Heatstroke Experience						*p*-Value	
		No		Yes					
		N	(%)	n	(%)	n	(%)		
Question		224	(60.4)	147	(39.6)	371	(100.0)		
Wear moisture-wicking clothing	Yes	151	(59.4)	103	(40.6)	254	(68.5)	0.590	
	No	73	(62.4)	44	(37.6)	117	(31.5)		
Wear cooling fabric underwear	Yes	115	(55.3)	93	(44.7)	208	(56.1)	0.024	*
	No	109	(66.9)	54	(33.1)	163	(43.9)		
Wear clothing with cooling features (air conditioning, cooling packs)	Yes	61	(59.2)	42	(40.8)	103	(27.8)	0.778	
	No	163	(60.8)	105	(39.2)	268	(72.2)		
Change clothes frequently	Yes	76	(56.3)	59	(43.7)	135	(36.4)	0.224	
	No	148	(62.7)	88	(37.3)	236	(63.6)		
Hat modifications	Yes	19	(73.1)	7	(26.9)	26	(7.0)	0.170	
	No	205	(59.4)	140	(40.6)	345	(93.0)		
Use of towels or similar items to shield face and neck from direct sunlight	Yes	38	(61.3)	24	(38.7)	62	(16.7)	0.872	
	No	186	(60.2)	123	(39.8)	309	(83.3)		
Wear long-sleeved clothing	Yes	33	(66.0)	17	(34.0)	50	(13.5)	0.382	
	No	191	(59.5)	130	(40.5)	321	(86.5)		
Wear clothing made from UV-protective materials	Yes	20	(58.8)	14	(41.2)	34	(9.2)	0.846	
	No	204	(60.5)	133	(39.5)	337	(90.8)		
Wear arm sleeves or arm covers	Yes	24	(60.0)	16	(40.0)	40	(10.8)	0.959	
	No	200	(60.4)	131	(39.6)	331	(89.2)		
Frequent hydration	Yes	207	(58.8)	145	(41.2)	352	(94.9)	0.008	**
	No	17	(89.5)	2	(10.5)	19	(5.1)		
Consume salt directly (e.g., pickled plums, salted candy, tablets) or through sports drinks, etc.	Yes	164	(59.4)	112	(40.6)	276	(74.4)	0.521	
	No	60	(63.2)	35	(36.8)	95	(25.6)		
Apply cold items to the head or neck	Yes	74	(54.8)	61	(45.2)	135	(36.4)	0.098	
	No	150	(63.6)	86	(36.4)	236	(63.6)		
Use of sunscreen	Yes	41	(53.9)	35	(46.1)	76	(20.5)	0.199	
	No	183	(62.0)	112	(38.0)	295	(79.5)		
Reduce alcohol consumption the previous day	Yes	15	(71.4)	6	(28.6)	21	(5.7)	0.286	
	No	209	(59.7)	141	(40.3)	350	(94.3)		
Simultaneous hydration and salt intake	Yes	209	(60.2)	138	(39.8)	347	(93.5)	0.826	
	No	15	(62.5)	9	(37.5)	24	(6.5)		
Health status check	Yes	106	(57.0)	80	(43.0)	186	(50.1)	0.181	
	No	118	(63.8)	67	(36.2)	185	(49.9)		
Use of mist showers and mist fans	Yes	94	(64.4)	52	(35.6)	146	(39.4)	0.204	
	No	130	(57.8)	95	(42.2)	225	(60.6)		
Utilize heat index (WBGT)	Yes	33	(58.9)	23	(41.1)	56	(15.1)	0.810	
	No	191	(60.6)	124	(39.4)	315	(84.9)		
Heat acclimatization	Yes	91	(63.6)	52	(36.4)	143	(38.5)	0.309	
	No	133	(58.3)	95	(41.7)	228	(61.5)		
Lower body temperature before activity (Precooling)	Yes	35	(54.7)	29	(45.3)	64	(17.3)	0.306	
	No	189	(61.6)	118	(38.4)	307	(82.7)		
(Countermeasure) Unknown	Yes	220	(60.8)	142	(39.2)	362	(97.6)	0.323	
	No	4	(44.4)	5	(55.6)	9	(2.4)		
Experience viewing the Ministry of Health, Labour and Welfare’s Heatstroke Prevention Manual	Yes	20	(57.1)	15	(42.9)	35	(9.4)	0.681	
	No	204	(60.7)	132	(39.3)	336	(90.6)		
Experience receiving education or guidance on heatstroke countermeasures	Yes	72	(57.6)	53	(42.4)	125	(33.7)	0.436	
	No	152	(61.8)	94	(38.2)	246	(66.3)		
Warnings or advice from supervisors regarding extreme heat during work	Yes	118	(61.1)	75	(38.9)	193	(52.0)	0.755	
	No	106	(59.6)	72	(40.4)	178	(48.0)		

** *p* < 0.01; * *p* < 0.05.

**Table 4:** Relationship between heatstroke experience and living conditions/health status.

		Heatstroke Experience						*p*-Value	
		No		Yes					
		n	(%)	n	(%)	n	(%)		
Question		224	(60.4)	147	(39.6)	371	(100.0)		
A. BMI_1	Normal (between 18 and <25)	171	(64.0)	96	(36.0)	267	(72.0)	0.026	*
	Underweight (<18.5)	19	(61.3)	12	(38.7)	31	(8.4)		
	Obese (≥25)	34	(46.6)	39	(53.4)	73	(19.7)		
B. BMI_2	Normal, Underweight (below 18.5)	190	(63.8)	108	(36.2)	298	(80.3)	0.007	**
	Obese (≥25)	34	(46.6)	39	(53.4)	73	(19.7)		
C. BMI_3	Normal, Obese (≥25)	205	(60.3)	135	(39.7)	340	(91.6)	0.914	
	Underweight (<18.5)	19	(61.3)	12	(38.7)	31	(8.4)		
D. Number of meals per day	3 times/4 times or more	150	(61.2)	95	(38.8)	245	(66.0)	0.642	
	None/1 time/2 times	74	(58.7)	52	(41.3)	126	(34.0)		
E. Number of days eating breakfast	0: Daily/4–5 days per week/2–3 days per week	163	(59.5)	111	(40.5)	274	(73.9)	0.557	
	Rarely eat	61	(62.9)	36	(37.1)	97	(26.1)		
F. Breakfast portion size	Large	9	(45.0)	11	(55.0)	20	(5.4)	0.150	
	Just right	88	(63.8)	50	(36.2)	138	(37.2)		
	Less than average	93	(63.3)	54	(36.7)	147	(39.6)		
	Do not eat	34	(51.5)	32	(48.5)	66	(17.8)		
G. Meal size (Lunch)	Large	37	(59.7)	25	(40.3)	62	(16.7)	0.126	
	Just right	154	(62.9)	91	(37.1)	245	(66.0)		
	Less than average	26	(47.3)	29	(52.7)	55	(14.8)		
	Do not eat	7	(77.8)	2	(22.2)	9	(2.4)		
H. Meal size (Dinner)	Large	109	(59.6)	74	(40.4)	183	(49.3)	0.721	
	Just right	108	(62.1)	66	(37.9)	174	(46.9)		
	Less than average	6	(54.5)	5	(45.5)	11	(3.0)		
	Do not eat	1	(33.3)	2	(66.7)	3	(0.8)		
I. Frequency of meals with staple food, main dish, and side dish	1 time/Rarely	100	(56.5)	77	(43.5)	177	(47.7)	0.258	
	2 times	80	(62.0)	49	(38.0)	129	(34.8)		
	3 times	44	(67.7)	21	(32.3)	65	(17.5)		
J. Sleep duration	7 h or more	65	(65.7)	34	(34.3)	99	(26.7)	0.210	
	Less than 7 h	159	(58.5)	113	(41.5)	272	(73.3)		
K. Satisfaction with sleep	Yes	74	(71.8)	29	(28.2)	103	(27.8)	0.005	**
	No/Neither	150	(56.0)	118	(44.0)	268	(72.2)		
L. Perceived health	Yes	121	(74.2)	42	(25.8)	163	(43.9)	<0.001	**
	No/Neither	103	(49.5)	105	(50.5)	208	(56.1)		
M. Alcohol consumption	No	109	(58.0)	79	(42.0)	188	(50.7)	0.338	
	Yes/No response	115	(62.8)	68	(37.2)	183	(49.3)		
N. Smoking	No	117	(60.6)	76	(39.4)	193	(52.0)	0.920	
	Yes/Past smoker	107	(60.1)	71	(39.9)	178	(48.0)		
O. Hypertension	No	215	(61.1)	137	(38.9)	352	(94.9)	0.234	
	Yes	9	(47.4)	10	(52.6)	19	(5.1)		
P. Dyslipidemia	No	215	(60.6)	140	(39.4)	355	(95.7)	0.730	
	Yes	9	(56.3)	7	(43.8)	16	(4.3)		
Q. Anemia	No	214	(61.5)	134	(38.5)	348	(93.8)	0.087	
	Yes	10	(43.5)	13	(56.5)	23	(6.2)		
R. Illness or treatment	No	188	(62.5)	113	(37.5)	301	(81.1)	0.089	
	Yes	36	(51.4)	34	(48.6)	70	(18.9)		
S. Medication use	No	199	(62.0)	122	(38.0)	321	(86.5)	0.107	
	Yes	25	(50.0)	25	(50.0)	50	(13.5)		

** *p* < 0.01; * *p* < 0.05.

**Table 5:** Odds ratios for physical symptoms, preventive measures, health status, etc., related to heatstroke experience.

Item	Odds Ratio	Confidence Interval		*p*-Value	
		Lower Limit	Upper Limit		
Physical symptoms of headache ^a^	2.657	1.253	5.636	0.011	*
Physical symptoms of dizziness ^a^	2.061	1.118	3.800	0.021	*
Wearing cooling fabric underwear ^b^	0.590	0.370	0.939	0.026	*
Frequent hydration ^b^	0.187	0.041	0.863	0.032	*
BMI ^c^	1.857	1.056	3.267	0.032	*
Self-perceived health ^d^	2.194	1.355	3.551	0.001	**

** *p* < 0.01; * *p* < 0.05; ^a^: None at all = 0, Rarely/Sometimes/Often = 1; ^b^: Yes = 0, No = 1; ^c^: Normal (between 18 and <25)/Underweight (<18.5) = 0, Obese (≥25) = 1; ^d^: Yes = 0, No/Neither = 1.

## Data Availability

The data presented in this study are available on request from the corresponding author due to privacy and ethical constraints.

## Supplementary Materials

The following supporting information can be downloaded at https://www.mdpi.com/article/10.3390/healthcare14101293/s1, Table S1: All variables, corresponding survey items, response options, and coding procedures used in the analysis.
