# Supplementary material for: Heatstroke Awareness and Preventive Behaviors Among Automotive Maintenance Workers in Outdoor Environments: A Cross-Sectional Study in Japan
Source: Healthcare (Basel). 2026 May 10;14(10):1293. doi: 10.3390/healthcare14101293 (PMC13205164; doi:10.3390/healthcare14101293)
Supplement: Supplementary file 1 [file healthcare-14-01293-s001.zip › healthcare-4260997-supplementary.pdf]

Table S1. All variables, corresponding survey items, response options, and coding procedures used in the analysis.

Variable	Survey Item	Response Options	Coding
Heatstroke experience	Have you ever experienced heatstroke during work activities?	Yes/No	Yes = 1, No = 0
Headache	Do you experience headache during summer work?	Not at all/Rarely/Sometimes/ Often	None at all = 0, Rarely/Sometimes/Often = 1
Dry mouth	Do you experience dry mouth during summer work?	Not at all/Rarely/Sometimes/ Often	None at all = 0, Rarely/Sometimes/Often = 1
Dizziness	Do you experience dizziness during summer work?	Not at all/Rarely/Sometimes/ Often	None at all = 0, Rarely/Sometimes/Often = 1
Fatigue/Weakness	Do you experience fatigue or weakness during summer work?	Not at all/Rarely/Sometimes/ Often	None at all = 0, Rarely/Sometimes/Often = 1
Difficulty concentrating	Do you experience difficulty concentrating during summer work?	Not at all/Rarely/Sometimes/ Often	None at all = 0, Rarely/Sometimes/Often = 1
Nausea	Do you experience nausea during summer work?	Not at all/Rarely/Sometimes/ Often	None at all = 0, Rarely/Sometimes/Often = 1
Muscle cramps	Do you experience muscle cramps during summer work?	Not at all/Rarely/Sometimes/ Often	None at all = 0, Rarely/Sometimes/Often = 1
Abnormally rapid breathing	Do you experience abnormally rapid breathing during summer work?	Not at all/Rarely/Sometimes/ Often	None at all = 0, Rarely/Sometimes/Often = 1
Rapid and weak pulse	Do you experience rapid and weak pulse during summer work?	Not at all/Rarely/Sometimes/ Often	None at all = 0, Rarely/Sometimes/Often = 1
Numbness in the lips	Do you experience numbness in the lips during summer work?	Not at all/Rarely/Sometimes/ Often	None at all = 0, Rarely/Sometimes/Often = 1
Abnormal speech and behavior	Do you experience abnormal speech or behavior during summer work?	Not at all/Rarely/Sometimes/ Often	None at all = 0, Rarely/Sometimes/Often = 1
Fainting	Do you experience fainting during summer work?	Not at all/Rarely/Sometimes/ Often	None at all = 0, Rarely/Sometimes/Often = 1
Hallucinations	Do you experience hallucinations during summer work?	Not at all/Rarely/Sometimes/ Often	None at all = 0, Rarely/Sometimes/Often = 1
Wear moisture-wicking clothing	Do you wear moisture-wicking clothing during summer work?	Yes/No	Yes = 0, No = 1
Wear cooling fabric underwear	Do you wear cooling fabric underwear during summer work?	Yes/No	Yes = 0, No = 1
Frequent hydration	Do you practice frequent hydration during summer work?	Yes/No	Yes = 0, No = 1

BMI	Body mass index calculated from self-reported height and weight	Underweight (<18.5)/Normal (18.5–<25)/Obese (≥25)	Normal/Underweight = 0, Obese = 1
Sleep satisfaction	Are you satisfied with your sleep?	Yes/No/Neither	Yes = 0, No/Neither = 1
Perceived health	Do you perceive yourself as healthy?	Yes/No/Neither	Yes = 0, No/Neither = 1

Heatstroke experience was assessed by a single item asking, ‘Have you ever experienced heatstroke during work activities?’ (Yes/No). Participants were asked to respond based on their own subjective judgment, without requiring a formal medical diagnosis. No specific clinical criteria were provided to participants. This self-reported measure may therefore capture a broader spectrum of heat-related symptoms beyond medically confirmed heatstroke.
